# Accuracy of Machine Learning Models to Predict Mortality in COVID-19 Infection Using the Clinical and Laboratory Data at the Time of Admission

**DOI:** 10.7759/cureus.18768

**Published:** 2021-10-14

**Authors:** Mohsen Tabatabaie, Amir Hossein Sarrami, Mojtaba Didehdar, Baharak Tasorian, Omid Shafaat, Houman Sotoudeh

**Affiliations:** 1 Health Information Management, Arak University of Medical Sciences, Arak, IRN; 2 Radiology, University of Semnan, Semnan, IRN; 3 Medical Parasitology and Mycology, Arak University of Medical Sciences, Arak, IRN; 4 Internal Medicine, Arak University of Medical Sciences, Arak, IRN; 5 Radiology and Radiological Science, Johns Hopkins University School of Medicine, Baltimore, USA; 6 Radiology, University of Alabama at Birmingham School of Medicine, Birmingham, USA

**Keywords:** pandemics, prognosis, mortality, covid-19, artificial intelligence, machine learning

## Abstract

Aim

This study aimed to develop a predictive model to predict patients’ mortality with coronavirus disease 2019 (COVID-19) from the basic medical data on the first day of admission.

Methods

The medical data including the demographic, clinical, and laboratory features on the first day of admission of clinically diagnosed COVID-19 patients were documented. The outcome of patients was also recorded as discharge or death. Feature selection models were then implemented and different machine learning models were developed on top of the selected features to predict discharge or death. The trained models were then tested on the test dataset.

Results

A total of 520 patients were included in the training dataset. The feature selection demonstrated 22 features as the most powerful predictive features. Among different machine learning models, the naive Bayes demonstrated the best performance with an area under the curve of 0.85. The ensemble model of the naive Bayes and neural network combination had slightly better performance with an area under the curve of 0.86. The models had relatively the same performance on the test dataset.

Conclusion

Developing a predictive machine learning model based on the basic medical features on the first day of admission in COVID-19 infection is feasible with acceptable performance.

## Introduction

In December 2019, a mass outbreak of coronavirus occurred in Wuhan, China. The World Health Organization (WHO) formally named the disease coronavirus disease 2019 (COVID-19) on February 11, 2020. Because of the rapid spread of the virus, there has been a sharp rise in the demand for medical resources to support infected people [1]. In about 20% of the patients, the infection is severe and may necessitate hospitalization. A mortality rate of about 13.4% has been reported in the severe form of the disease [2,3]. The risk assessment and knowing the predictors of death among patients diagnosed with COVID-19 is crucial to target high-risk patients through early and more intensive interventions [4]. In addition to efficient diagnosis and treatment, accurate prognosis prediction is necessary to reduce the strain on healthcare systems and provide the best possible care for patients. When allocating limited medical resources, prediction models that estimate the risk of a poor outcome in an infected individual based on pre-diagnosis information could help to triage patients more effectively [5].

So far, several predictive models for COVID-19 have been used, and it appears that the most important medical features are age, body temperature, gender, creatinine level, and blood pressure [6]. However, designing a standard study about prognosis prediction is challenging. Many studies suffer from weak design and likely overestimation of the model performance, as was evident in a systematic review by Wynants and colleagues. They reported that most predictive models are prone to high bias, mainly because of selection bias, unclear reporting, and a high chance of overfitting [6].

Machine learning (ML), a branch of artificial intelligence (AI) that learns from past data to build predictive models, can help in this circumstance [7]. In recent years, ML has been developed as a useful tool to analyze large amounts of data from medical records or medical images [8]. Recently, there are advances in the prediction of COVID-19 using ML. These advances include estimating the mortality risk in patients with suspected or confirmed COVID-19, predicting progression to a severe or critical state, and predicting the hospital stay duration [9].

This study aimed to develop an ML platform for outcome prediction in admitted COVID-19 patients. We try to develop clinical and laboratory features accessible in small rural medical centers and not the sophisticated ones, including computed tomography (CT) images.

## Materials and methods

This study was approved by the ethical committee (Arak University of Medical Sciences) of our university. Informed consent was obtained from all subjects upon admission.

Retrospective part of the study: training dataset

The electronic medical records of the patients admitted to a tertiary referral hospital from March 20, 2020, to November 20, 2020, were evaluated retrospectively. All adult patients (age above 18 years) with a clinical diagnosis of COVID-19 infection were included. The clinical diagnosis was consistent with the physician’s diagnosis based on the patients’ exposure to positive cases of COVID-19 and positive clinical and imaging findings. The polymerase chain reaction (PCR) test was not used as a part of the inclusion criteria. However, this test was used as one of the predictive features to predict mortality. The patients who left the hospital against the physicians’ recommendation were excluded. All patients with a clinical diagnosis of COVID-19 who died during the admission were recorded. The patients admitted and discharged with the clinical COVID-19 infection during the same time interval were more than the expired patients. However, only 270 discharged patients were included randomly to avoid class imbalance.

Multiple medical features (predictive features) related to the patients’ demographic, medical history, and clinical and laboratory findings “at the time of admission” were extracted and documented (Table 1). The outcome (target features) was considered as discharge or death. The mentioned features were analyzed by the Orange data mining platform version 3.27 (Bioinformatics Lab at University of Ljubljana, Slovenia) [10]. Upon the feature selection, the most important predictive features were selected. Subsequently, different ML models were trained based on the selected features to predict the outcome (discharge versus death). The performance (sensitivity, specificity, accuracy, and area under the curve {AUC}) of each model was then evaluated and reported by 10-fold cross-validation. Moreover, different ensemble models were developed by combining variable ML models. The performances of the ensemble models were also assessed and reported by 10-fold cross-validation. 

**Table 1:** List of demographics, medical history, clinical, and laboratory features used in this study. PCR: polymerase chain reaction; O_2_sat: O2 saturation; BS: blood sugar; BUN: blood urea nitrogen; Cr: creatinine; Na: sodium; K: potassium; Ca: calcium; CPK: creatine phosphokinase; LDH: lactate dehydrogenase; SGPT: alanine aminotransferase; SGOT: aspartate aminotransferase; Alk-P: alkaline phosphatase; WBC: white blood cell; RBC: red blood cell; Hb: hemoglobin; Hct: hematocrit; MCV: mean corpuscular volume; MCH: mean corpuscular hemoglobin; MCHC: mean corpuscular hemoglobin concentration; ESR: erythrocyte sedimentation rate; PT: prothrombin time; PTT: partial thromboplastin time; INR: international normalized ratio; HCO_3_: bicarbonate; CRP: C-reactive protein

Demographic feature	Clinical feature	History of comorbidities	Laboratory feature
Age and sex	Time between initial symptoms and admission; time between initial symptoms and the first PCR test, O_2_sat; respiratory rate per minute; pulse rate per minute; temperature; blood pressure, systolic; blood pressure, diastolic; dyspnea; cough; sputum; weakness; headache; vertigo; altered mental status; anosmia; chills; diarrhea; nausea; vomiting; anorexia, myalgia; chest pain; and arthralgia	Hypertension; smoker/addiction; chronic lung diseases; diabetes mellitus; chronic kidney diseases; heart diseases; cancer; and rheumatologic diseases	First PCR; BS; BUN; Cr, Na; K; Ca; CPK; LDH; SGPT; SGOT; Alk-P; total bilirubin; direct bilirubin; WBC; RBC; Hb; Hct; MCV; MCH; MCHC; platelet; lymphocyte; monocyte; eosinophil; basophil; ESR; PT; PTT; INR; PH; PCO_2_; base excess; base excess in the extracellular fluid compartment; HCO_3_; and CRP

Prospective part of the study: test dataset

Upon compilation of the study, the final trained models were tested on a test dataset. For the test dataset, the patients with the clinical diagnosis of the COVID-19 who were admitted to the same hospital were included prospectively from November 21, 2020, to February 3, 2021. Upon evaluating the medical record, the demographic, medical history, and clinical and laboratory data at the time of admission were collected in the same fashion as described in the training dataset. All consecutive patients in the mentioned time window were included. The patients who left the hospital against the physicians' recommendation were excluded. The data collection did not interfere with patients management. The physicians and nurses who took care of the patients were not informed. The trained models were then tested on this prospective dataset to predict the outcome. The performance of each model was then calculated from the confusion matrix.

All statistical analyses were performed using SPSS version 22 (Chicago, IL: SPSS Inc.) and R version 3.3.3 (Vienna, Austria: R Foundation for Statistical Computing). The P-value of 0.05 was considered as the cutoff point.

## Results

After implementing the inclusion and exclusion criteria, 520 patients were included in this project with a mean age of 67.48 years and a standard deviation (SD) of 16.27; 264 patients (50.77%) were female (Table 2). The included patients consist of 270 patients discharged after hospitalization and 250 patients who died during the hospitalization. The patients discharged after hospitalization consists of 121 (44.81%) males and 149 (55.18%) females with a mean age of 62.18 years and SD of 16.58. The patients who died during hospitalization consist of 135 (54%) males and 115 (46%) females with a mean age of 73.2 years and SD of 13.83. The mean age of these two discharged and expired cohorts was significantly different (P-value=0.001).

**Table 2:** The demographic of patients within the training dataset.

Outcome		Sex		Age	
		Number	Percent	Mean (years)	Standard deviation
Discharged	Male	121	44.8	61.3884	16.59989
	Female	149	55.2	62.8389	16.59211
Expired	Male	135	54	75.4074	14.13913
	Female	115	46	70.6261	13.07298
P-value		0.043		0.001	

After implementing the “Info. Gain” as the feature selection model, and using the cutoff point of 0.02, 22 out of 70 features were selected as the most predictive features (Table 3).

**Table 3:** List of the selected features used in this study. O_2_sat: O2 saturation; N/A: not applicable; PCR: creatine phosphokinase; BS: blood sugar; BUN: blood urea nitrogen; Cr: creatinine; LDH: lactate dehydrogenase; SGOT: aspartate aminotransferase; WBC: white blood cell

Demographic feature	Clinical feature	Comorbidities	Laboratory feature
Age	Time between initial symptoms and admission; O_2_sat; respiratory rate; temperature; dyspnea; nausea; and myalgia	N/A	First PCR; BS; BUN; Cr; LDH; SGOT; total bilirubin; direct bilirubin; WBC; monocyte; eosinophil; base excess; base excess in the extracellular fluid compartment; and HCO_3_

Different ML models were trained on top of these 22 features to predict patients' mortality or discharge outcomes. From different ML models (e.g., k-nearest neighbors, decision tree, random forest, support vector machine, naive Bayes, AdaBoost, and neural network), the naive Bayes demonstrated the best performance with an AUC of 0.85, an accuracy of 80%, the sensitivity of 78% and specificity of 82%. The ensemble model from the naive Bayes and neural network combination had slightly better performance with an AUC of 0.86, an accuracy of 80%, a sensitivity of 80%, and a specificity of 80% (Table 4).

**Table 4:** The performance of trained machine learning models on the train and test datasets. AUC: area under the curve; SVM: support vector machine; RF: random forest; DT: decision tree; KNN: k-nearest neighbors; NB: naive Bayes; AdaBoost: adaptive boosting

Models	Sensitivity in train group-test group (%)	Specificity in train group-test group (%)	Accuracy in train group-test group (%)	AUC in train group-test group
Ensemble (NB +neural network)	80-85	80-61	80-81	0.86-0.85
NB	78-81	82-76	80-80	0.85-0.82
Neural network	78-82	75-61	76-79	0.84-0.81
AdaBoost	72-76	73-76	72-76	0.72-0.76
SVM	70-79	76-69	73-77	0.80-0.82
RF	75-75	72-69	73-74	0.82-0.78
DT	68-67	67-69	68-68	0.66-0.66
KNN	73-62	56-53	65-61	0.68-0.6

The test dataset consists of 94 patients with the clinical diagnosis of COVID-19. There were 44 (46.81%) males and 50 (53.19%) females, with a mean age of 59.45 years (SD of 17.64). Eighty-one patients were discharged and 13 patients died during hospitalization. The trained models were then tested on the test dataset. Again, the naive Bayes had the best performance with an AUC of 0.82, an accuracy of 80%, a sensitivity of 81%, and a specificity of 76%. The ensemble model from the naive Bayes and neural network combination had slightly better performance with an AUC of 0.85, an accuracy of 81%, a sensitivity of 85%, and a specificity of 61% (Table 4). 

## Discussion

Prognosis prediction is critical for the triage of patients with COVID-19. In this context, the ML-based models appear promising. For instance, Vaid et al. implemented the Extreme Gradient Boosting (XGBoost) on medical records [11]. They could predict the mortality on days three, five, and seven after hospitalization with an AUC of 0.89, 0.85, and 0.84, respectively. In another AI model by a convolutional neural network (CNN), researchers differentiated the progressive form of infection versus the non-progressive form using CT images and clinical data with an AUC of 0.91 [12].

In this study, we trained various ML models to obtain the best performance for mortality prediction of patients with COVID-19 infection based on clinical and laboratory findings on the first day of admission. We tried to select the training dataset in a way that does not suffer from class imbalance. By the feature selection, we also tried to avoid overfitting. The trained models were then tested on the test dataset to make sure there is no substantial overfitting. 

In our study, the most important features in terms of the prediction power were saturation of O_2_ (O_2_sat), lactate dehydrogenase (LDH), age, blood urea nitrogen (BUN), base excess, creatinine, base excess in the extracellular fluid compartment, white blood cell (WBC) count, HCO_3_, the result of PCR test, blood sugar, bilirubin total, myalgia, monocyte count, respiratory rate, bilirubin direct, the time between initial symptoms and admission (days), nausea, temperature, eosinophil count, Aspartate aminotransferase (SGOT), and dyspnea.

Several of our selected features are common among other published researches. Pan et al. evaluated 100 medical features to predict the mortality in 123 intensive care unit (ICU) admitted COVID-19 patients. They found out that the following eight features are significantly associated with mortality: lymphocyte percentage, prothrombin time, lactate dehydrogenase, total bilirubin, eosinophil percentage, creatinine, neutrophil percentage, and albumin level. An XGBoost model based on these features had an AUC of 0.92 for mortality prediction [13]. Ma et al. have reported the LDH, C-reactive protein (CRP), and age as the most important predictive features for mortality; their multivariate logistic regression model had the AUC of 0.95 for mortality prediction [14]. A random forest developed by Parchure et al. on 55 final medical features had an AUC of 0.85 in mortality prediction within 20-84 hours from the time of prediction [15]. By evaluation of the medical data from 1270 COVID-19 patients, Guan et al. found the following six predictive features to predict the mortality in COVID-19: age, levels of high-sensitivity C-reactive protein (hs-CRP), LDH, ferritin, and interleukin-10 (IL-10). The subsequent Simple-tree XGBoost model predicted the mortality with accuracy above 90% [16]. In a nationwide cohort of COVID-19 in South Korea, the researchers evaluated the data from 10,237 patients. They reported the age above 70 years, male sex, moderate or severe disability, being in a nursing home, having diabetes mellitus, chronic lung disease, and asthma as significant predictors of mortality. They reported the AUC of 0.96 for least absolute shrinkage and selection operator (LASSO) and linear support vector machine (SVM) models using these features [5].

By evaluating the studies mentioned above, it seems that the LDH level is a common predictive feature in most of them, including our study. However, there is significant variation among other selected features. This difference can be partial because of the demographic differences between these studies (e.g., different ethnicities, mean age, access to medical services, etc.).

There is controversy about the role of comorbidities in predictive models about COVID-19. In our study, the comorbidities were not found to be significantly predictive. Several previously published studies emphasized the role of comorbidities in the prediction of mortality in COVID-19 patients. For instance, Wollenstein-Betech et al. trained different models based on demographic data and comorbidities of symptomatic COVID-19 patients. In their study, SMV and LR showed the best performance to predict the hospitalization, mortality, need for ICU, and need for ventilator with the accuracy of 72%, 79%, 89%, and 90%, respectively [17]. In another study, hypertension as the most common comorbidity in COVID-19 patients had a controversial effect on severe illness and mortality [18]. In a meta-analysis by Silverio et al., hypertension did not show an independent contribution regarding the in-patient mortality rate [19]. However, in some other surveys, hypertension showed a strong correlation with the severity and fatality of COVID-19 [20]. Diabetes and dyslipidemia had predictive value for severe illness and mortality in different studies [20-23].

Our study has several limitations. The laboratory data are fairly accurate; however, the clinical data (e.g, vital signs) were documented from the medical records. The healthcare workers in our medical center did not receive additional training for our study. They were blind to the study, thus, the recorded clinical data can be influenced by the staff experience and accuracy. The other limitation would be the lack of imaging biomarkers that were previously revealed to have predicting value [24,25]. However, we tried to focus on easily accessible medical data that are feasible in small medical centers. There are many other factors that could interfere with the predictive potency of our findings, such as the therapeutic strategy for each patient, underlying deficiency of vitamins/minerals [26,27], interleukin levels [28], and the different virus mutants/variants [29,30]. In addition, our study is different than most similar researches. In most studies, the PCR test was used as an inclusion criterion for COVID-19 infection. However, in this study, the patients with a "clinical diagnosis" of COVID-19 who underwent standard of care treatment were included. In this context, the PCR test was used as a predictive feature rather than an inclusion criterion. This strategy was selected to address the false-negative PCR tests. Finally, this study has the intrinsic limitations of machine learning especially the concept of the black box; the processes of decision making and prognosis prediction are not transparent in these models. Although we tried to avoid the overfitting by the feature selection, all patients included in this study belong to a specific region, and it is unclear if the final models work well for other ethnicities.

## Conclusions

Prediction of the mortality from the basic medical data on the first day of admission is feasible using the ML models with acceptable accuracy. Such models may end in better patient triage and improve these patients' survival by referring the high-risk cases to well-equipped centers at the time of admission. In our study, the naive Bayes model showed the best performance for this task. The selected features in our research are accessible medical data and are feasible to be recorded in small rural clinics. In the future, these models' performance may be improved by adding sophisticated medical data and images from tertiary medical centers.
